# Draft Genome Sequence of the Opportunistic Marine Pathogen *Vibrio harveyi* Strain E385

**DOI:** 10.1128/genomeA.00677-13

**Published:** 2013-12-12

**Authors:** Mingjia Yu, Chunhua Ren, Jinrong Qiu, Peng Luo, Ruyi Zhu, Zhe Zhao, Chaoqun Hu

**Affiliations:** Engineering Research Center of Marine Biological Resource Comprehensive Utilization, Third Institute of Oceanography, State Oceanic Administration, Xiamen, Chinaa; CAS Key Laboratory of Tropical Marine Bio-resources and Ecology, South China Sea Institute of Oceanology, Chinese Academy of Sciences, Guangzhou, Chinab; South China Institute of Environmental Sciences, MEP, Guangzhou, Chinac

## Abstract

*Vibrio harveyi* strain E385 was isolated from a diseased cage-cultured grouper in Daya Bay, China. Phylogenetic analysis based on the 16S rRNA gene sequence showed similarity with *V. harveyi* strain BAA-1116. We sequenced the pathogenic strain *V. harveyi* E385 and compared the genome with that of the nonpathogenic strain *V. harveyi* BAA-1116.

## GENOME ANNOUNCEMENT

*Vibrio harveyi* is a Gram-negative halophilic bacterium that can be found swimming free in tropical marine waters as part of the resident microflora of marine animals. It has also been recognized as an opportunistic pathogen to many commercially farmed marine invertebrate and vertebrate species (1). *V. harveyi* strain E385, isolated from a diseased cage-cultured grouper (*Epinephelus coioides*) at the Daya Bay of China in 2009, has been identified as a member of a *V. harveyi* clade and shares a high degree of genetic similarity with *V. harveyi* strain BAA-1116. Importantly, the microorganism is a virulent strain, based on the results of artificial infection tests.

*V. harveyi* E385 was sequenced on the HiSeq 2000 sequencing platform with a paired-end 2 × 100-nucleotide (nt) procedure. A total of 24,892,942 paired-end reads were generated, with an average length of 100 bp. After adapter and quality trimming of these data, there was about 2.4 Gb of filtered or clean data remaining, which yielded almost 400-fold coverage. *De novo* assembly was performed through the CLC Genomics Workbench (2) and SeqMan (3), resulting in the production of 94 scaffolds ranging from 551 bp to 765,364 bp. Excluding the gaps, the draft genome has a total of 6,354,192 bp, with a G+C content of 44.8%.

Using the draft genome, protein-coding sequences were predicted using the Glimmer 3.02 program (4), and 5,639 predicted open reading frames (ORFs) were obtained. These predicted ORFs were annotated by a BLASTx search against the SwissProt database with a cutoff *E* value of 10^-10^. In total, 3,652 ORFs were significantly matched by BLASTx hits, covering 64.8% of all predicted ORFs. Furthermore, GO annotation was analyzed using the Blast2GO software (5, 6), by which GO terms were assigned to query sequences and catalogued groups were produced based on biological processes, molecular functions, and cellular components. In total, 2,857 ORFs were assigned with 14,313 GO terms. Within molecular functions, catalytic activity categories (GO:0003824) and binding (GO:0005488) were highly represented, accounting for 41.3% and 34.8% of the ORFs, respectively. Cells (GO:0005623; 45.7%) and cell parts (GO:0044464; 45.7%) were the most represented GO categories within the cellular components. As to biological processes, metabolic process (GO:0008152; 28.9%) was the most highly represented category, followed by cellular process (GO:0009987; 26.1%). Using KEGG analysis (7) with the bidirectional best hit (BBH) method, 114 pathways were mapped, including metabolic pathways (127 members, KEGG:ko01100), followed by secondary metabolite biosynthesis (64 members, KEGG:ko01110), microbial metabolism in diverse environments (37 members, KEGG:ko01120), ABC transporters (27 members, KEGG:ko02010), two-component system (26 members, KEGG:ko02020), and flagellar assembly (15 members, KEGG:ko02040). To classify the possible functions of the genes, COG annotation was performed (8), and the results showed that 177 and 99 of the predicted proteins fall into the U class (intracellular trafficking, secretion, and vesicular transport) and V class (defense mechanisms), respectively. Noncoding RNA genes were identified by tRNAscan-SE (9) and RNAmmer (10) and found 19 16S-23S-5S rRNA and 105 predicted tRNA genes in the genome. Interestingly, we also found 85 toxin genes via an online prediction tool (see http://mvirdb.llnl.gov/) (11), with the parameters of the filter for BLAST results chosen using the default settings. These results emphasize its high virulence toward marine hosts.

### Nucleotide sequence accession numbers.

This whole-genome shotgun project has been deposited at DDBJ/EMBL/GenBank under the accession no. AYKI00000000. The version described in this paper is version AYKI01000000.
